# Pseudorabies virus tegument protein US2 antagonizes antiviral innate immunity by targeting cGAS-STING signaling pathway

**DOI:** 10.3389/fimmu.2024.1403070

**Published:** 2024-07-02

**Authors:** Zhengjie Kong, Xing Chen, Lele Gong, Lele Wang, Yifeng Zhang, Kaifeng Guan, Wanzi Yao, Yu Kang, Xinyi Lu, Yuhang Zhang, Yongkun Du, Aijun Sun, Guoqing Zhuang, Jianguo Zhao, Bo Wan, Gaiping Zhang

**Affiliations:** ^1^ School of Advanced Agricultural Sciences, Peking University, Beijing, China; ^2^ Key Laboratory of Animal Immunology, Henan Academy of Agricultural Sciences, Zhengzhou, China; ^3^ State Key Laboratory of Stem Cell and Reproductive Biology, Institute of Zoology, Chinese Academy of Sciences, Beijing, China; ^4^ International Joint Research Center of National Animal Immunology, College of Veterinary Medicine, Henan Agriculture University, Zhengzhou, China; ^5^ Longhu Laboratory, Henan Agricultural University, Zhengzhou University, Zhengzhou, China

**Keywords:** pseudorabies virus, cGAS-STING, tegument protein US2, TRIM21, immune invasion

## Abstract

**Background:**

The cGAS-STING axis-mediated type I interferon pathway is a crucial strategy for host defense against DNA virus infection. Numerous evasion strategies developed by the pseudorabies virus (PRV) counteract host antiviral immunity. To what extent PRV-encoded proteins evade the cGAS-STING signaling pathway is unknown.

**Methods:**

Using US2 stably expressing cell lines and US2-deficient PRV model, we revealed that the PRV tegument protein US2 reduces STING protein stability and downregulates STING-mediated antiviral signaling.

**Results:**

To promote K48-linked ubiquitination and STING degradation, US2 interacts with the LBD structural domain of STING and recruits the E3 ligase TRIM21. TRIM21 deficiency consistently strengthens the host antiviral immune response brought on by PRV infection. Additionally, US2-deficient PRV is less harmful in mice.

**Conclusions:**

Our study implies that PRV US2 inhibits IFN signaling by a new mechanism that selectively targets STING while successfully evading the host antiviral response. As a result, the present study reveals a novel strategy by which PRV evades host defense and offers explanations for why the Bartha-K61 classical vaccine strain failed to offer effective defense against PRV variant strains in China, indicating that US2 may be a key target for developing gene-deficient PRV vaccines.

## Introduction

The innate immunity is the host’s first line of defense against pathogen invasion. After viral infection, host cells recognize molecular patterns associated with the pathogen that are structurally consistent, which prompts them to quickly start a series of signaling processes that result in the production of type I interferon (IFN) and other antiviral substances (1). After sensing viral DNA in the cytoplasm, cGAS catalyzes the formation of cyclic GMP-AMP (cGAMP) from ATP and GTP (2). cGAMP further activates STING, a crucial node protein on the endoplasmic reticulum (3). In microsomes, activated STING translocates away from the endoplasmic reticulum, recruits the partner molecule TBK1, and phosphorylated TBK1 recruits IRF3 (4). Activated IRF3 translocates from the cytoplasm to the nucleus to initiate the production of type I IFN and induce antiviral immune responses (5, 6).

The zoonotic disease pseudorabies, resulting from the pseudorabies virus (PRV), is one of the most hazardous outbreaks endangering the further growth of swine farming (7). Pseudorabies virus is also known as porcine herpesvirus, and pigs are natural containers of PRV (8). PRV can infect different age groups of pigs, resulting in reproductive disorders, abortion, stillbirth in sows, neurological disorders and death in piglets, sterility in breeding boars, and immunocompromise and growth retardation in fattening pigs (9). PRV can infect many mammals, causing morbidity or acute death in humans, domestic animals, dogs, and mice (10–12). The study of the PRV pathogenic mechanism is of major importance to the prevention and management of animal diseases and the health and safety of people due to the large variety of hosts that PRV may infect and its high pathogenicity.

Herpesviruses are a class of immunosuppressive viruses encoding viral proteins that can modulate the immune response and promote viral immune escape in different ways (13, 14). PRV, a member of the α-herpesvirus family, has evolved strategies to antagonize host immune responses (15). It has been reported that various tegument proteins encoded by PRV may regulate antiviral innate immunity mediated by the cGAS-STING signaling pathway, thereby promoting viral replication and latent infection (16). PRV tegument protein UL21 binds cGAS and induces cGAS degradation via the selection autophagy pathway (17). PRV UL13 targets STING and IRF3 and inhibits the activation of DNA signaling pathways (18, 19). The proteasome route degrades Bclaf1 due to PRV US3, and ISGF3’s ability to bind to ISRE is also prevented (20). PRV UL42 competitively binds ISRE with ISGF3 and reduces the production of ISGs (21). These reports suggest that PRV tegument proteins can inhibit host immune responses in several ways. However, more mechanisms by which PRV escapes host innate immunity and regulates the type I IFN response remain unclear.

Our study demonstrated that the PRV tegument protein US2 functions as a new regulator of the cGAS-STING pathway, preventing IFN production and antiviral immunity in response to PRV infection. While US2 interacts with STING and reduces its stability, US2 deficiency reduces the amount of STING protein degraded due to PRV. Particularly, US2 engages the E3 ubiquitin ligase TRIM21, which encourages K48-linked ubiquitination and STING degradation, preventing STING from mediating type I IFN and downstream ISG production. Our study thus reveals a novel strategy and mechanism by which PRV US2 controls STING stability by enlisting TRIM21 to circumvent STING-mediated antiviral immune reactions.

## Materials and methods

### Mice

Female C57BL/6 mice were acquired from Liaoning Changsheng Biotechnology Co., Ltd. at the age of six weeks, and they were cared for in a particular pathogen-free facility following the guidance for the handling and use of laboratory animals. The Henan Agricultural University Animal Care and Use Committee approved the mouse research (approval code: HNND2023092601). Mice were intraperitoneally injected with 0.2 ml of 5×10^5^ PFU of PRV-WT or PRV-ΔUS2 (n = 5 in each group) for the survival assay (22). Each group’s mice were tracked for how long they survived. The mouse serum was collected at 1 dpi. At 4 days post-infection, infected mice’s lungs and brains were taken and either viral load or H&E-stained.

### Cells and viruses

Hela cells, 3D4/21 cells, HEK293T cells, PK-15 cells, and BHK-21 cells were from the American Type Culture Collection (ATCC, VA, USA). Cells were grown in DMEM (Gibco, USA) supplemented with 10% fetal bovine serum (FBS) and 1% penicillin-streptomycin from Gibco at 37°C in 5% CO_2_. Mycoplasma tests on all cells returned negative results. The International Joint Research Center of National Animal Immunology (Zhengzhou, China) donated PRV (HeNZZ strain), replicated in BHK-21 cells.

### Plasmids

PrimeStar HS DNA Polymerase (Takara) was used to amplify the PRV US2 gene from the genomic DNA of the PRV HeNZZ strain (GenBank: OQ744679.1) and clone it into pCMV-Myc and pLOV expression vector. Amplification of TRIM21, STING, and its truncation mutants (amino acids 1-139, 140-380, 140-340, 180-340, and 180-380) from the cDNA of Hela cells was performed. Ubiquitin plasmids (WT, K48, and K48R) and STING plasmids were kindly provided by International Joint Research Center of National Animal Immunology (Zhengzhou, China).

### US2 stable cell line generation

The stable cell lines stabling expressing US2 or its truncated mutants were established using the lentivirus-mediated gene-editing technology (23). Briefly, HEK293T cells were transfected with the packaging plasmids psPAX2 and pMD2.0G, recombinant plasmids, or the empty vector pLOV. After 36–48 hours, the pseudovirus-containing culture supernatant was harvested to infect PK-15 cells in the presence of polybrene (8 µg/mL). The infected cells were screened with puromycin (3 µg/mL) for 6 days to establish stable cell lines.

### Antibodies and reagents

The following are the sources and antibodies that were used: Cell Signaling Technology sold the following antibodies: cGAS (79978), p-TBK1 (5483), TBK1 (3013), and p-IRF3 (4947). Proteintech Group Inc. sold the following antibodies: STING (19851-1-AP), His tag (10001-0-AP), HA tag (51064-1-AP), and β-actin (66009-1-lg). Santa Cruz Biotechnology provided the Protein A/G PLUS-Agarose beads (sc-2003), c-Myc antibody (sc-40), and IRF3 antibody (sc-33641). We bought anti-Flag M2 Affinity Gel (A2220) and anti-Flag (F1804) antibody from Sigma. Abcam sold the anti-TRIM21 antibody (ab91423). Both cGAMP (tlrl-nacga 23-02) and poly(dA:dT) (tlrl-patn) were bought from Invivogen. Thermos Fisher Scientific provided the transfection agent Lipofectamine 2000. From Solarbio Life Sciences, Opti-MEM, protease inhibitors, and PMSF were acquired. PRV gE antibody was kindly gifted by Prof. Junqing Guo (Henan Academy of Agricultural Sciences).

### Daul-luciferase reporter assay

Using Lipofectamine 2000, the IFN-β or ISRE luciferase reporter plasmids, renilla luciferase reporter, US2, or empty control vectors were co-transfected into PK-15 cells. Cells were harvested for luciferase assay 24 h after transfection or stimulated with poly(dA:dT) (1 µg/ml) for 12 h. The Dual-Luciferase Report Assay System (Promega, USA) was used to produce and analyze cell lysates following the manufacturer’s guidelines.

### Generation of PRV-ΔUS2 using CRISPR-Cas9

The procedures previously outlined were used to create the PRV-ΔUS2 recombinant strain. The gRNA website (https://zlab.bio/guide-design-resources) was used to build two gRNAs manufactured by Sango Biotech in Shanghai, China, targeting the PRV US2 gene. Two pX459 plasmids were ligated with two US2 gRNAs. The plasmids pX459-US2-gRNA1 and pX459-US2-gRNA2 were co-transfected into HEK293T cells for 24 h. PRV HeNZZ was used to infect cells for 24 h (MOI=1). The virus mixture was gathered, and the plaque test was performed. After DNA sequencing, monoclonal viruses were detected by PCR. The target sequences for US2 gRNA are as follows: #1 5’-CAGGATCCACAGGTGGA-3’; #2 5’-CAGGGCCTCCGTCCACTCG C-3’. In PK-15 cells, all viruses were multiplied and titrated according to established techniques.

### Co-immunoprecipitation and immunoblotting

As previously mentioned, immunoprecipitation and immunoblotting were carried out (18). Plasmids were briefly transfected into the appropriate cells by Lipofectamine 2000. Cells were lysed on ice with Triton lysis buffer for 60 min 24 h after transfection. Extracts were immunoprecipitated with 1 µg of the designated antibodies and protein A/G PLUS-Agarose beads. Specific antibodies were used to perform immunoblotting.

### Reverse transcription and quantitative real-time PCR

Using the M-MLV reverse transcriptase with RNase inhibitor from Takara, total RNA was isolated using the SV Total RNA Isolation System from Promega. Viral DNA was isolated per the directions using a DNA isolation kit (TIANGEN). DNA from each sample was mean normalized. SYBR Green Master Mix (Vazyme) was used in triplicate for qPCR on an ABI QuantStudio5 instrument. qPCR was used to quantify viral genomic DNA using primers for the PRV gD gene. Glyceraldehyde-3-phosphate dehydrogenase (GAPDH)-specific primers were used to amplify triplicated samples, and threshold cycle numbers were then standardized to those values. The **Supplementary Table S1** contains a list of qPCR primer sequences.

### Immunofluorescence

The indicated plasmids were transfected into Hela cells, and after 20 min, the cells were fixed in 4% paraformaldehyde. The cells were then blocked with 5% bovine serum albumin (Gibco) for 30 min after being permeabilized with 0.1% Triton X-100 on ice for 10 min. Alexa Fluor 488 or 594-conjugated secondary antibodies (Proteintech Group Inc.) were used to stain the cells after they had been treated with the appropriate primary antibodies for 2 h. A 63-oil objective Zeiss confocal microscope was used to capture the images. The Pearson’s correlation coefficient (PCC) was used to indicate the co-localization between TRIM21 (red) and Flag-US2 (green) by Image J.

### LC-MS/MS

HEK293T cells were co-transfected with the expression plasmids for Flag-STING and Myc-US2 or control. After 24 h of transfection, the cells were collected, and the lysates were immunoprecipitated using anti-Flag M2 Affinity Gel. The eluted samples were washed, resolved using SDS-PAGE, and stained with Coomassie brilliant blue. The Jingjie PTM BioLab (Hangzhou) Co. Inc. conducted an LC-MS/MS study (**Supplementary Table S2**).

### RNA interference

Shanghai GenePharma Co., Ltd., a Chinese company, created US2 siRNA oligos. Sango Biotech previously reported on and produced human TRIM21 siRNA (24). Following the manufacturer’s recommendations, Lipofectamine RNAiMAX (Invitrogen) reagent transfected siRNA oligos into HEK293T cells for 24 h at a final concentration of 50 nM. The US2 siRNA oligos are: #1 5’-UCACCGUGGUCACGCUGAUTT-3’, #2 5’-UCUACAUCACCACCGAGACTT -3’, #3 5’-GGGAUCCAGUACUGAACUTT-3’.

### Generation of TRIM21 knockout cells

TRIM21 gRNAs were created by Sango Biotech using the gRNA website (https://zlab.bio/guide-design-resources) to produce TRIM21 knockout cells. The lentiCRISPRv2 plasmid was ligated to the gRNA sequence. Two packaging plasmids (psPAX2 and pVSVG), an empty vector, and lentiCRISPRv2-TRIM21 plasmid were co-transfected into HEK293T cells. After 48 h, the recombinant viruses were filtered, polybrene was added (8 µg/ml, Cat#H8641, Solarbio), and 3D4/21 cells were infected. Puromycin (Cat#IP1160, Solarbio) was used to select cells for 3 days at a final 4 µg/ml concentration. 96-well plates were used to generate monoclonal cells identified by immunoblotting with the TRIM21 antibody. The TRIM21 gRNA target sequence is 5’- GAACCTCCGGCCCAATCGGC-3’.

### Enzyme-linked immunosorbent assay

IFN-β secretion in serum was quantified by ELISA according to the manufacturer’s instructions (18). Anti-mouse IFN-β antibodies (Capture antibody, 22400-1; Detection antibody, 32401-1) were bought from PBL. Mouse IFN-β (12405-1, PBL) was used as a protein standard.

### Histopathology

The procedure for histopathology was as previously mentioned (10). Briefly, mouse brain or lung tissues were embedded in paraffin after being fixed overnight in 10% neutral-buffered formalin and trimmed. Hematoxylin-eosin (H&E) staining was applied after sections were cut into 5 m. Images were captured using imaging software (CellSens Standard) using an Olympus microscope (CKX53).

### Statistical analysis

*P*-values were determined using the Prism GraphPad software (v8.0) and a two-tailed paired or unpaired Student’s *t*-test. *P*-values of 0.05 or lower were regarded as significant. The log-rank test was used to examine the statistical significance of the mice survival study.

## Results

### US2 inhibits STING-mediated signaling

**Figure 1:**
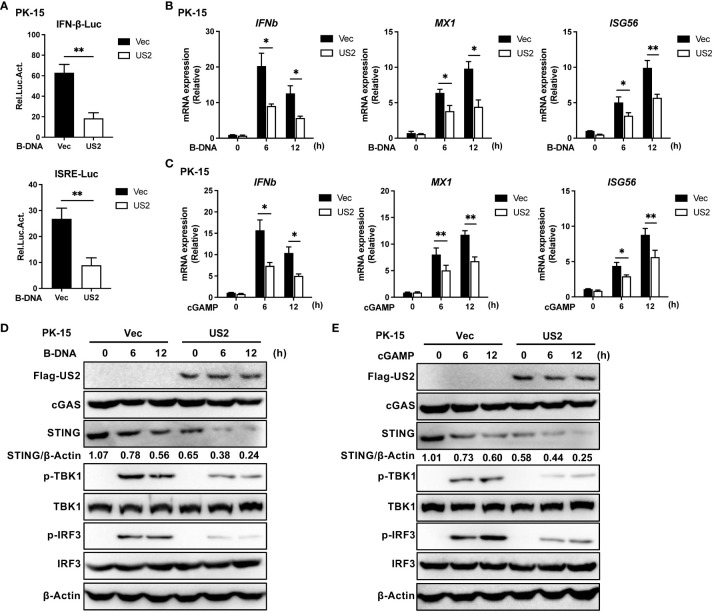
PRV US2 inhibits cGAS-STING signaling pathway-mediated antiviral immune responses. **(A)** PK-15 cells co-transfected with the US2 and IFN-Luc or ISRE-Luc vectors were stimulated with B-DNA (1 µg/ml) for 12 h. A dual-luciferase assay was applied to detect the IFN and ISRE promoters’ activity. **(B, C)** US2-PK-15 cells and control cells were transfected with B-DNA or cGAMP (1 µg/ml). qPCR study of B-DNA (1 µg/ml)- or cGAMP (1 µg/ml)-stimulated *IFNb1* and downstream *MX1* and *ISG56* mRNA expression. **(D, E)** US2-PK-15 cells and control cells were stimulated at various times with B-DNA (1 µg/ml) or cGAMP (1 µg/ml), respectively, cGAS, STING, phosphorylated (Ser172)- and total TBK1, and phosphorylated (Ser396)- and total IRF3 were analyzed by immunoblotting in **(D, E)**. The ratio of STING to β-Actin was analyzed by Image J. Data are pooled from three independent experiments [**(A–C)**, mean ± SD] or representative of two independent experiments **(D, E)**. *P < 0.05, **P < 0.01 (Student’s *t*-test).

Innate immunity plays a pivotal role in defense against herpesvirus infection. To determine the function of the PRV tegument protein US2 in evading the cGAS-STING signaling pathway, the impact of US2 on the activity of IFNβ and ISRE promoters was investigated using a luciferase assay. As a result of B-DNA Poly (dA:dT) stimulation in PK-15 cells revealed that US2 significantly suppressed the activities of IFN and ISRE promoters (**Figure 1A**), demonstrating that US2 functions as a type I IFN antagonist. To learn more about how US2 affects the expression of IFN, we established stable PK-15 cells ectopically expressing US2 (US2-PK-15 cells) by lentiviral-mediated transduction. In comparison to control cells, US2 stable expressing cells displayed reduced *IFNb1*, downstream *MX1*, and *ISG56* expression in response to B-DNA stimulation (**Figure 1B**). The adaptor protein STING can be activated further by cGAMP, which is produced when cGAS is activated (25). We looked at the impact of US2 on cGAMP-mediated innate immunity to determine whether US2 targets the cGAS-STING signaling pathway. Similar results were observed when *IFNb1*, *MX1*, and *ISG56* transcription was induced by cGAMP in PK-15 cells (**Figure 1C**), suggesting that US2 may target STING to block DNA-mediated immune responses. In the meantime, US2 prevented TBK1 and IRF3 from becoming phosphorylated in response to B-DNA or cGAMP (**Figures 1D, E**), indicating that US2 inhibits the cGAS-STING-mediated signaling pathway. Although TBK1 and IRF3 phosphorylation brought on by B-DNA or cGAMP stimulation was suppressed by US2 expression, US2 did not change the total protein of cGAS, TBK1, and IRF3. Notably, the production of US2 greatly decreased the levels of STING protein (**Figures 1D, E**), indicating that US2 likely inhibits STING-mediated innate immunity by concentrating on the expression of STING protein. When considered as a whole, these results show that PRV tegument protein US2 negatively affects the DNA signaling pathway-mediated antiviral immune response by concentrating on STING protein.

### US2 reduction enhances innate antiviral response to PRV

**Figure 2:**
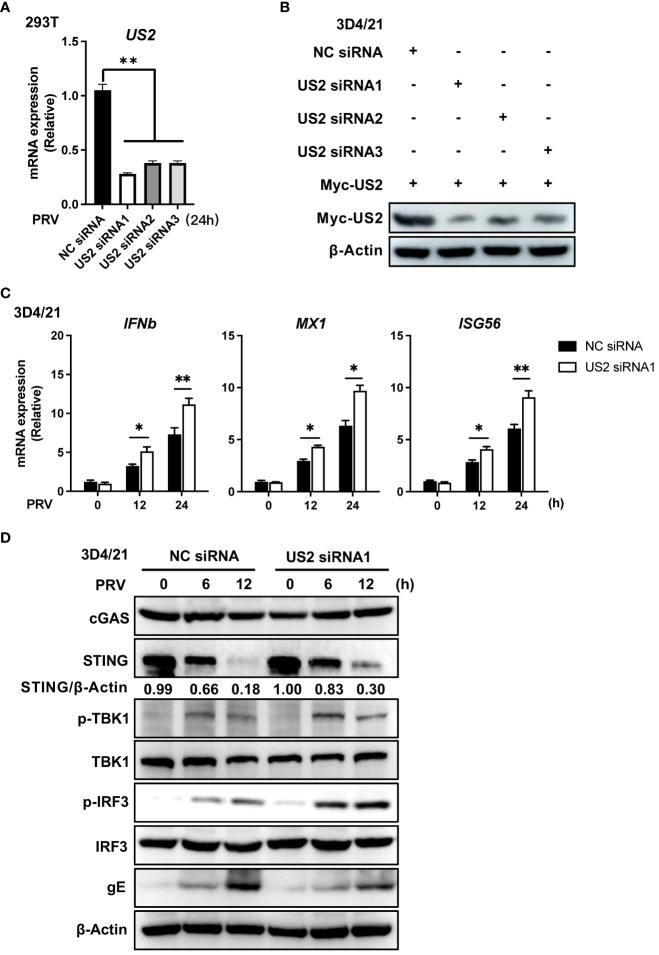
Knockdown of US2 increases PRV-triggered antiviral responses. **(A)** US2 siRNA and control siRNA were transfected into HEK293T cells, and 24 h later, the cells were exposed to PRV (MOI=1) for 24 (h) Analysis of US2 gene transcription by qPCR in cells infected with PRV. **(B)** Immunoblotting examination of the expression of US2 protein in 3D4/21 cells that were co-transfected for 24 h with US2 and US2 siRNAs or control siRNA. **(C)** US2 siRNA and control siRNA were transfected into 3D4/21 cells, and 24 h later, the cells were infected for 24 h with PRV (MOI=1). In cells infected with PRV (MOI=1) at the specified times, qPCR analysis of PRV-induced transcription of *IFNb1*, *MX1*, and *ISG56* was performed. **(D)** Immunoblotting examination of cGAS, STING, and the phosphorylation of downstream components in 3D4/21 cells transfected with US2 siRNA or NC siRNA and exposed to PRV (MOI=1) for the specified durations. The ratio of STING to β-Actin was analyzed by Image J. Data are pooled from three independent experiments [**(A, C)**, mean ± SD] or representative of two independent experiments **(B, D)**. *P< 0.05, **P< 0.01 (Student’s *t*-test).

We then looked into the impact of endogenous US2 on the host antiviral immune response since it blocks DNA-triggered production of IFN and downstream antiviral genes. We created various interfering RNAs that prevented the expression of the US2 gene and protein (**Figures 2A, B**). A model of endogenous US2 knockdown viral infection was formed by ectopically expressing US2 siRNA in cells, which were then infected with PRV. *IFNb1*, *MX1*, and *ISG56* transcription were upregulated by PRV in US2-knockdown cells compared to control siRNA-transduced cells (**Figure 2C**), indicating that blocking US2 improved the antiviral responses that PRV elicited. Similar to this, the reduction of US2 increased the phosphorylation of TBK1 and IRF3, the downstream components of STING, at various times following PRV infection and stabilized STING protein levels (**Figure 2D**). The total amount of TBK1 and IRF3 was unaffected by US2 interference, nor were the amounts of the cGAS protein (**Figure 2D**). These findings imply that lowering STING protein levels and raising PRV-induced antiviral immune responses may be accomplished by decreasing US2 expression.

### US2-deficiency heightens innate antiviral response to PRV

**Figure 3:**
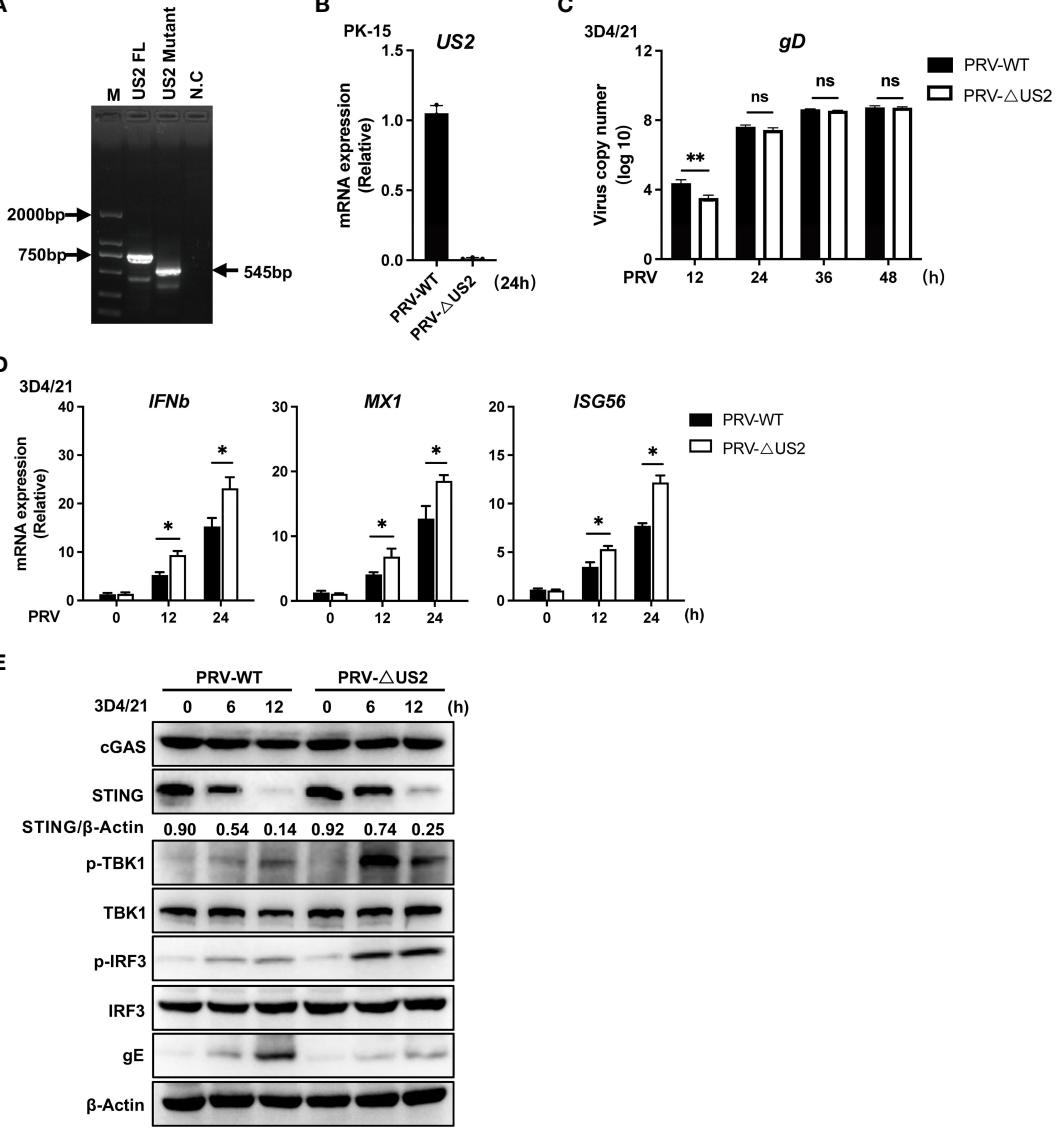
Deficiency of US2 increases PRV-triggered antiviral responses. **(A)** PCR examination of the PRV-WT and PRV-ΔUS2 US2 gene. **(B)** qPCR investigation of US2 gene transcription in cells that had been infected for 24 h with PRV-WT (MOI=1) or PRV-ΔUS2 (MOI=1). **(C)** qPCR analysis of replication of PRV-WT (MOI=1) or PRV-ΔUS2 (MOI=1) during the specified timeframes in 3D4/21 cells. **(D)** qPCR analysis of PRV-WT or PRV-ΔUS2-induced transcription of *IFNb1*, *MX1*, and *ISG56* for the respective times was performed on 3D4/21 cells following infection with PRV-WT (MOI=1) or PRV-ΔUS2 (MOI=1) for the specified timeframes. **(E)** Immunoblotting examination of cGAS, STING, and the phosphorylation of downstream components in 3D4/21 cells infected for the specified periods with PRV-WT (MOI=1) or PRV-ΔUS2 (MOI=1). The ratio of STING to β-Actin was analyzed by Image J. Data are pooled from three independent experiments **(B–D)**, mean ± SD) or representative of two independent experiments **(E)**. *P < 0.05, **P < 0.01, ns, not significant (Student’s *t*-test).

We created US2-deficient PRV using CRISPR/Cas9 technology to demonstrate further the regulatory role of endogenous US2. PCR analysis showed that the gene set of US2 was partially knocked out (**Figure 3A**) and US2 gene expression was not observed (**Figure 3B**). Meanwhile, we found that US2-deficient PRV replication was attenuated in 3D4/21 cells, suggesting that US2 affects PRV replication (**Figure 3C**). The expression of downstream antiviral genes in 3D4/21 cells stimulated by wild-type or US2-deficient PRV was then examined. The findings demonstrated that PRV-ΔUS2 greatly increased the gene expression of *IFNb1*, *MX1*, and *ISG56* in 3D4/21 compared to wild-type PRV (**Figure 3D**). Accordingly, TBK1 and IRF3 were considerably phosphorylated more by PRV-ΔUS2 than by wild-type PRV (**Figure 3E**). In addition, after infection with PRV-ΔUS2, the STING protein level declined more slowly than it did after infection with wild-type PRV (**Figure 3E**). These findings collectively imply that US2 directly contributes to PRV evasion of the innate antiviral response.

### US2 interacts with STING

**Figure 4:**
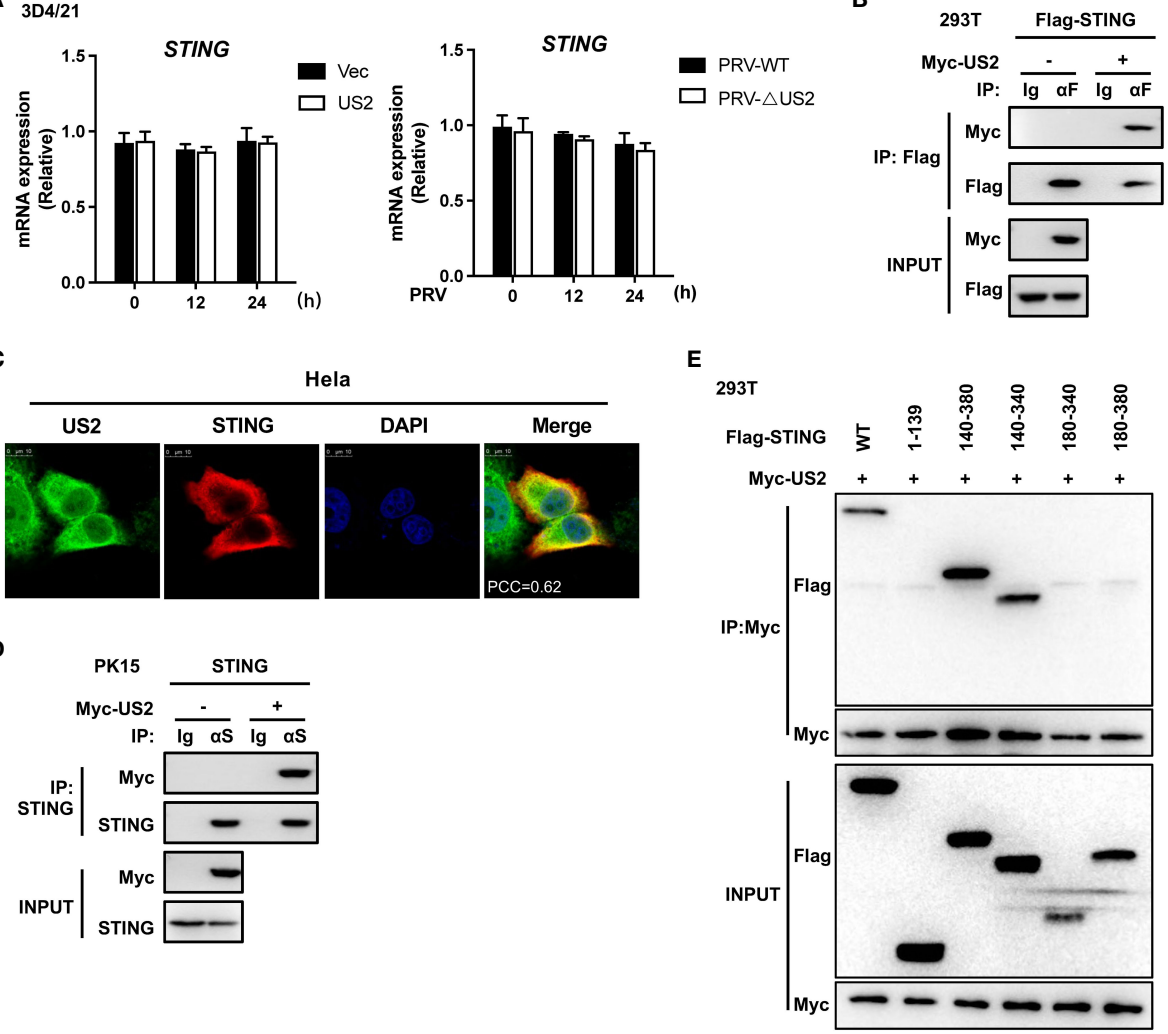
US2 associates with STING. **(A)** Left: Quantitative PCR measurement of STING gene expression in 3D4/21 cells expressing US2 or control vector cells. Right: qPCR study of STING gene expression in 3D4/21 infected with PRV-WT (MOI=1) or PRV-ΔUS2 (MOI=1) for the specified durations. **(B)** Analysis of the indicated proteins using immunoblotting (IB) in whole-cell lysates and immunoprecipitated (IP) samples from HEK293T cells transfected with Myc-US2 and Flag-STING. By using anti-Flag antibodies for immunoblotting, anti-Flag immunoprecipitations were examined. Anti-Flag or anti-Myc antibodies were used to measure the transfected proteins’ levels in immunoblotting. **(C)** Colocalization of STING and US2. Before confocal imaging, Hela cells were transfected for 24 h with Myc-US2 and Flag-STING as directed. The Pearson’s correlation coefficient (PCC) was used to indicate the co-localization between STING (red) and Flag-US2 (green) by Image J. **(D)** Anti-STING immunoprecipitants from PK-15 cells that expressed Myc-US2 were examined by immunoblotting using anti-STING antibodies. Using anti-Myc or anti-STING antibodies, immunoblotting was used to measure the transfected proteins’ levels. **(E)** Immunoblotting analysis of the proteins US2 and STING in immunoprecipitated samples or whole-cell lysates from HEK293T cells transfected with full-length or truncated Flag-US2 and Myc-US2, respectively. By immunoblotting with an anti-Myc or anti-Flag antibody, anti-Myc immunoprecipitants were examined. The transfected proteins’ levels were examined using an anti-Myc or anti-Flag antibody during immunoblotting.

We then looked at how US2 controls the STING protein because it decreases the expression of STING protein. First, we looked at how US2 affected the expression of the STING gene. US2 expression had little impact on STING mRNA expression compared to control cells (**Figure 4A**, left). Additionally, qPCR analysis revealed that US2 did not affect STING mRNA expression in 3D4/21 cells infected with PRV (**Figure 4A**, right). With this information in hand, our next goal was to determine if US2 regulates STING protein expression at the post-transcriptional level. According to transient transfection and co-precipitation assays, US2 was connected to STING in HEK293T cells (**Figure 4B**). Additionally, confocal microscopy examination revealed that US2 and STING co-localized in Hela cells (**Figure 4C**), indicating that STING can bind to US2. Immunoprecipitation tests revealed that US2 may bind to endogenous STING in PK-15 cells (**Figure 4D**), confirming the relationship between US2 and STING. STING (aa 140-340) was sufficient for the interaction, according to mapping tests of structural domains (**Figure 4E**) (3, 26), demonstrating that the binding requires the Ligand-binding domain (LBD) structural domain of STING. Together, these findings show that US2 and STING have direct interactions.

### US2 enhances K48-linked ubiquitination of STING

**Figure 5:**
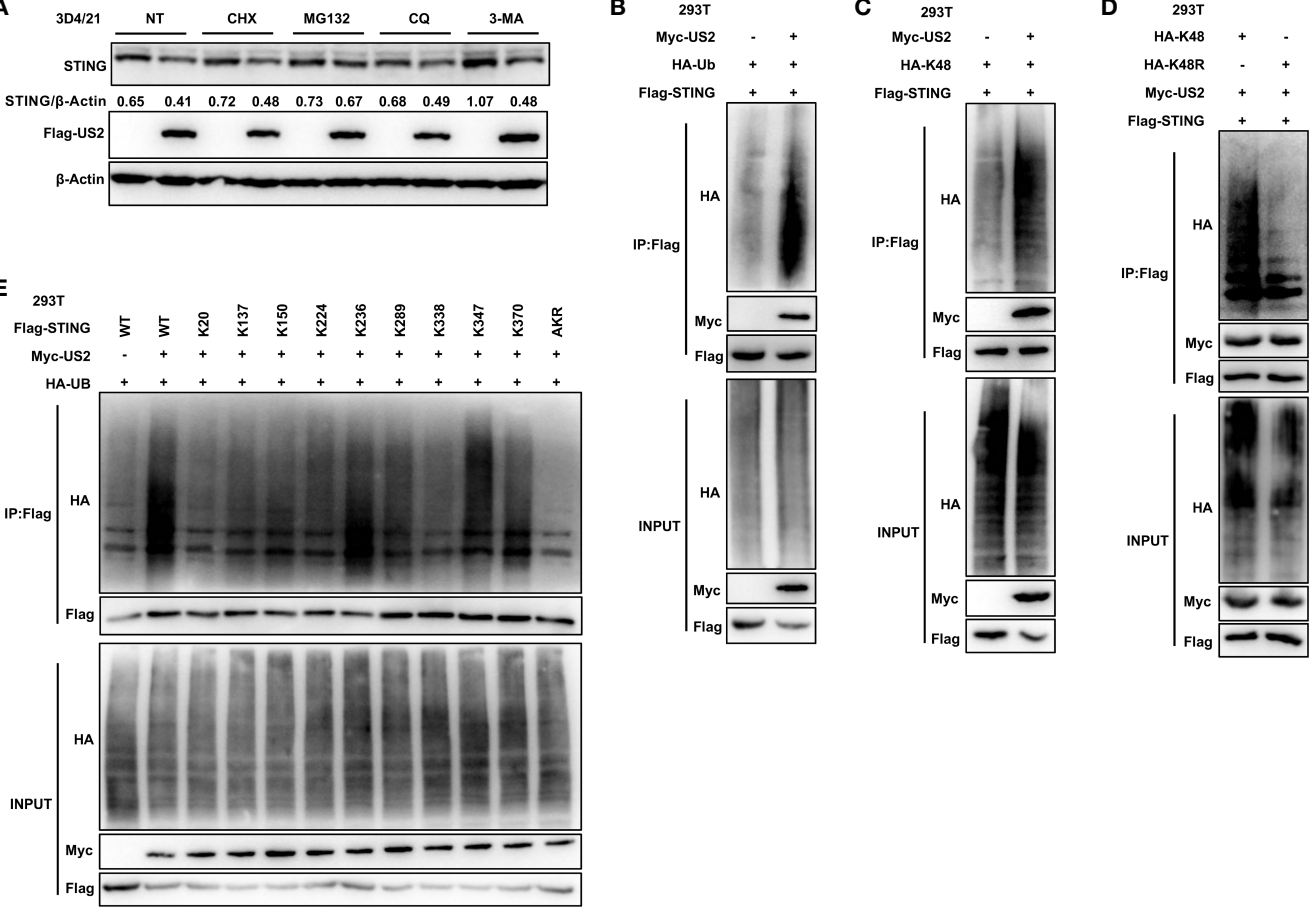
US2 targets STING for K48-linked ubiquitination and degradation. **(A)** Immunoblotting examination of the expression of the STING protein in US2 expression or control 3D4/21 cells after treatment with 10 µM concentrations of CHX, MG-132, CQ, or 3-MA for 8 hours, respectively. The ratio of STING to β-Actin was analyzed by Image J. **(B–D)** Immunoprecipitation study of HEK293T cells expressing HA-Ubiquitin (Ub) **(B)**, solely Lys-to-Arg mutant at position 48 (Ub-K48) **(C)**, or both (Ub-K48R) **(D)**, as well as Flag-STING with or without Myc-US2. **(E)** Immunoprecipitation study of HEK293T cells that express Flag-STING in combination with Myc-US2 and HA-Ub (wild type, STING-WT; K20, K137, K150, K224, K236, K289, K338, K347, and K370 alone; or Lys-to-Arg mutants of all STING lysines, STING-AKR) as indicated. Using an anti-Flag, anti-HA, or anti-Myc antibody, immunoblotting was used to examine anti-Flag immunoprecipitations. Using an anti-Flag, anti-HA, or anti-Myc antibody **(B–E)**, immunoblotting was used to measure the levels of the transfected proteins.

We sought to determine if and how US2 directly interacts with STING and influences STING protein levels. First, the protein synthesis inhibitor cycloheximide (CHX), the proteasome inhibitor MG132, or the lysosomal degradation pathway inhibitors Chloroquine (CQ) and 3-Methyladenine (3-MA) were applied to 3D4/21 cells. Comparatively, to cells transfected with an empty vector, cells transfected with US2 displayed a considerable reduction in STING protein levels in response to treatment with the protein synthesis inhibitor CHX. The proteasome inhibitor MG132 blocked STING degradation by US2. In contrast, treatment with the lysosomal inhibitors CQ and 3-MA did not retard STING degradation (**Figure 5A**), and these results suggest that US2 may decrease STING protein stability and promote STING degradation via the proteasome pathway. We then checked whether US2 impacts STING’s ubiquitination in HEK293T cells. It is clear that US2 targets STING for ubiquitination because the expression of US2 increases the ubiquitination of STING (**Figure 5B**). Considered to be one of the main signals for proteasome-mediated degradation is the K48-linked ubiquitin chain (27). So, our goal was to determine whether US2 encourages STING’s ubiquitination via the K48 pathway. We created this ubiquitin mutant (K48) that retains only the lysine residue at position 48. Results from immunoprecipitation demonstrated that US2 causes STING to be modified by K48 ubiquitination (**Figure 5C**). Additionally, we created ubiquitin mutants that had position 48 silenced (lysine was changed to arginine in place of position 48), and the results demonstrated that US2 was unable to facilitate the ubiquitination of STING (**Figure 5D**). According to these findings, US2 stimulates STING to create K48-linked ubiquitination modification. We created a variety of STING mutants with one lysine residue only at positions 20, 137, 150, 224, 236, 289, 338, 347, or 370 or with all lysine residues converted to arginine mutants (STING-AKR) to map the STING ubiquitination sites. Immunoprecipitation results showed that ubiquitination at 236, 347, or 370 was mostly triggered by US2, suggesting that the three lysines of STING (including K236, 347, and 370) are the major ubiquitination sites catalyzed by US2 (**Figure 5E**). These data demonstrate that US2 induces K48-linked polyubiquitination of STING, thereby inhibiting STING-mediated antiviral immunity.

### US2 recruits TRIM21 to initiate K48-linked ubiquitination of STING

**Figure 6:**
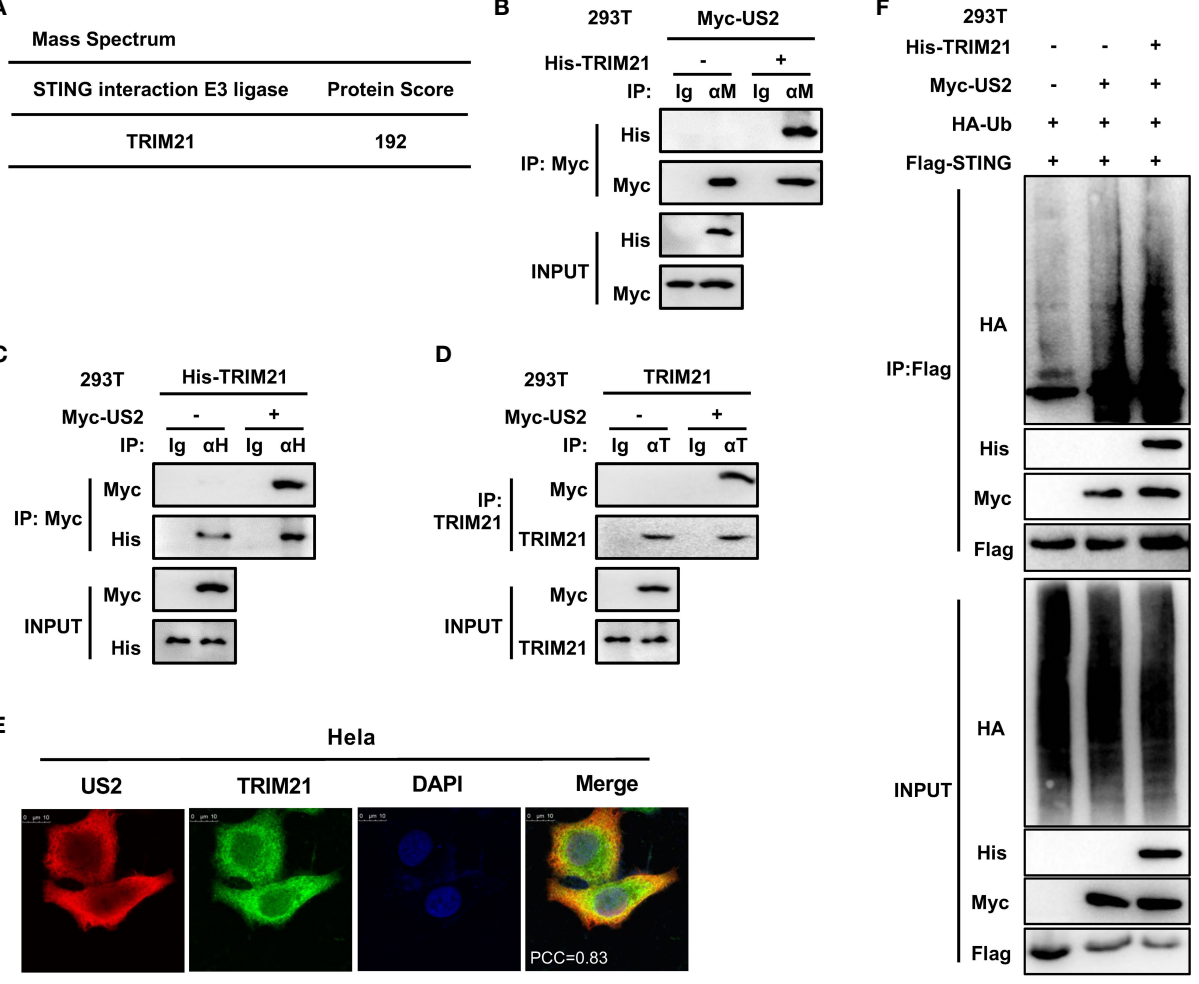
US2 recruits E3 ligase TRIM21 to induce STING ubiquitination degradation. **(A)** LC-MS examination of E3 ligase in immunoprecipitated HEK293T cells transfected with Flag-STING and Myc-US2 or control. **(B–D)** An immunoprecipitation study of HEK293T cells expressing either Myc-US2 alone **(D)** or Myc-US2 plus His-TRIM21 **(B, C)**. By immunoblotting with anti-His **(B)**, anti-Myc **(C, D)**, or anti-TRIM21 **(D)** antibodies, anti-Myc **(B)**, anti-His **(C)**, or anti-TRIM21 immunoprecipitants were examined. Immunoblotting was used to measure the levels of the transfected proteins using either anti-TRIM21 antibodies or anti-Myc and anti-His **(B, C)** antibodies. **(E)** Colocalization of TRIM21 and US2. Before confocal imaging, Hela cells were transfected for 24 h with Myc-US2 and His-TRIM21 as directed. The Pearson’s correlation coefficient (PCC) was used to indicate the co-localization between TRIM21 (red) and Flag-US2 (green) by Image J. **(F)** Immunoprecipitation analysis of Myc-US2, Flag-STING, and HA-Ub with or without His-TRIM21-expressing HEK293T cells. Anti-His, anti-Myc, and anti-HA antibodies were used to perform immunoblotting on anti-Flag immunoprecipitates. The transfected proteins’ levels were examined using anti-His, anti-HA, or anti-Myc antibodies in immunoblotting.

Since US2 causes STING to be degraded through ubiquitination, our next goal was to find any ubiquitin enzymes that might be implicated in this action. To do immunoprecipitation, we transfected Flag-STING and Myc-US2 into HEK293T cells. Only tripartite motif-containing protein 21 (TRIM21), a well-known E3 ligase, could be recruited to STING and US2 according to mass spectrometry analyses (**Figure 6A**). According to earlier research, TRIM21 regulates signaling pathways for cytoplasmic DNA recognition (28). As a result, we examined the connection between TRIM21 and US2. In HEK293T cells, immunoprecipitation tests suggested that US2 and TRIM21 may interact (**Figures 6B, C**). Also, US2 co-localized with TRIM21, suggesting that US2 might bind to TRIM21 (**Figure 6E**). Moreover, US2 interacted with endogenous TRIM21 (**Figure 6D**), suggesting that US2 targets the E3 ligase TRIM21. In addition, TRIM21 expression markedly promoted STING ubiquitination and degradation associated with US2 (**Figure 6F**). These findings show that US2 engages the E3 ligase TRIM21 to trigger the ubiquitination and degradation of STING.

### TRIM21 deficiency attenuates STING ubiquitination and enhances PRV-triggered antiviral responses

**Figure 7:**
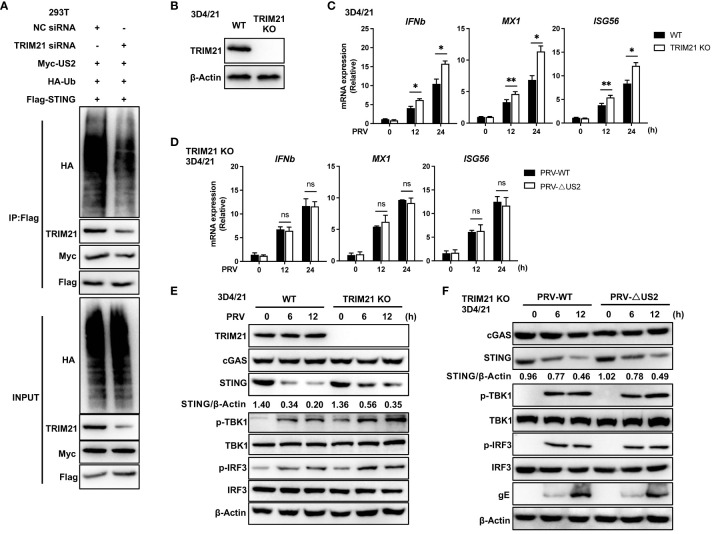
TRIM21-deficiency potentiates PRV-induced antiviral immune responses. **(A)** Control or TRIM21 siRNA (50 µM) was transfected into HEK293T cells. Cells were subsequently transfected for 24 h with Flag-STING, Myc-US2, and HA-Ub. Analysis using immunoblotting of the specified proteins in HEK293T cell lysates and immunoprecipitated samples. **(B)** Immunoblotting examination of the TRIM21 protein in 3D4/21 cells with wild-type (WT) and TRIM21-deficient (TRIM21 KO) genotypes. **(C)** Quantitative PCR study of *IFNb1*, *MX1*, and *ISG56* transcription in WT and TRIM21-deficient 3D4/21 cells infected with PRV (MOI=1) for the specified durations. **(D)** Quantitative PCR study of *IFNb1*, *MX1*, and *ISG56* transcription in TRIM21-deficient 3D4/21 cells infected with PRV-WT and PRV-ΔUS2 (MOI=1) for the specified durations. **(E)** Immunoblotting examination of the following proteins in whole-cell lysates of WT and TRIM21-deficient 3D4/21cells infected with PRV (MOI=1) for the given times: cGAS, STING, phosphorylated (Ser172) and total TBK1, phosphorylated (Ser396) and total IRF3, or TRIM21. **(F)** Immunoblotting examination of the following proteins in whole-cell lysates of TRIM21-deficient 3D4/21cells infected with PRV-WT and PRV-ΔUS2 (MOI=1) for the given times: cGAS, STING, phosphorylated (Ser172) and total TBK1, phosphorylated (Ser396) and total IRF3, or TRIM21. The ratio of STING to β-Actin was analyzed by Image J Data represent two independent experiments **(A, B, E, F)** or pooled from three independent experiments [**(C, D)**, mean ± SD]. *P< 0.05, **P< 0.01, ns, not significant (Student’s *t*-test).

Since TRIM21 expression catalyzes STING-associated ubiquitination with US2, we created human TRIM21-specific siRNA oligonucleotides (siTRIM21) to determine whether endogenous TRIM21 promotes STING ubiquitination (24). As anticipated, TRIM21 knockdown reduced the amount of STING ubiquitinated by US2 (**Figure 7A**), indicating that endogenous TRIM21 is the catalyst for STING ubiquitination. Due to the exceedingly low levels of STING expression in HEK293T cells (29), We subsequently examined whether endogenous TRIM21 controls the immunological response elicited by PRV in 3D4/21 cells. Following this, we generated a TRIM21-deficient cell model by CRISPR/Cas9 technology and confirmed TRIM21 deficiency by immunoblot analysis (**Figure 7B**). TRIM21 loss boosted PRV-induced production of *IFNb1*, *MX1*, and *ISG56* during PRV infection of cells (**Figure 7C**), elevated PRV-induced phosphorylation of TBK1 and IRF3, and slowed the degradation of endogenous STING (**Figure 7E**). Based on the above data, TRIM21 suppresses STING-mediated antiviral reactions when PRV is infected. Further, we infected TRIM21-deficient cell lines with two viruses. qPCR analysis showed that the induction of *IFNb* and downstream *ISG*s mediated by PRV-WT and PRV-ΔUS2 were similar in TRIM21 KO 3D4/21 cells (**Figure 7D**). Immunoblot analysis showed that two viruses induced similar phosphorylation levels of TBK1 and IRF3 and a consistent trend in the degradation of STING (**Figure 7F**). Our findings show how important STING degradation by TRIM21 is for the PRV-induced innate antiviral response.

### US2 promotes immune evasion of PRV *in vivo*

**Figure 8:**
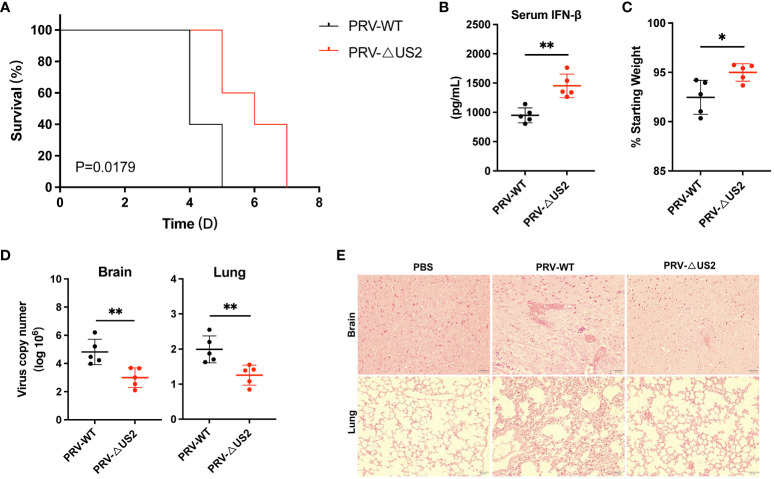
US2 deficiency enhances host defense against PRV infection *in vivo*. **(A)** The percentage of C57BL/6 mice that survived after receiving either a lethal dose of PRV-WT or PRV-ΔUS2 (5 × 10^5^ PFU, the same as below). **(B)** ELISA of IFN-β in serum from mice infected with PRV-WT or PRV-ΔUS2 (n = 5 in each group). Serum was collected at 1 dpi. **(C)** Body weight loss in PRV-WT or PRV-ΔUS2-infected mice at day 3. **(D)** qPCR investigation of viral replication at day 4 following infection with a dose of PRV-WT or PRV-ΔUS2 in mouse brain and lungs (n=5 in each group). **(E)** Mice’s brain and lung slices were stained with hematoxylin and eosin (H&E; 400x magnification). 50 μm scale bars. Data are one independent experiment [**(A–D)**, mean ± SD]. *P<0.05 and **P<0.01 (Student’s unpaired *t*-test or log-rank test in A).

We then examined the role of US2 in PRV immune evasion since it lowers immunological reactions. We created a model of PRV-infected mice for validation to comprehend the role of US2 in PRV pathogenicity *in vivo*. According to the findings, PRV-ΔUS2-infected mice outlived mice infected with wild-type PRV (**Figure 8A**), demonstrating that US2 deficiency promotes host defense against PRV. Serum levels of IFN-β in mice infected with PRV-ΔUS2 group were significantly higher than those in PRV-WT (**Figure 8B**). In contrast, mice infected with the PRV-ΔUS2 virus lost less weight than mice infected with the wild-type PRV (**Figure 8C**). Furthermore, compared to PRV-infected mice, PRV-ΔUS2-infected mice showed decreased viral copy counts in the brain and lungs (**Figure 8D**). Acute viral encephalitis with cerebral vascular congestion and bleeding, interstitial and hemorrhagic pneumonitis in the lungs, and other histological abnormalities were also present in mice infected with wild-type PRV. These findings indicate that the PRV-ΔUS2 virus is significantly less harmful in mice than those infected with PRV-WT, which led to the modest pathological alterations seen in PRV-ΔUS2-infected mice (**Figure 8E**). These findings show that US2 reduces the host’s ability to fight off a PRV infection and increases the pathogenicity of the virus *in vivo*.

## Discussion

**Figure 9:**
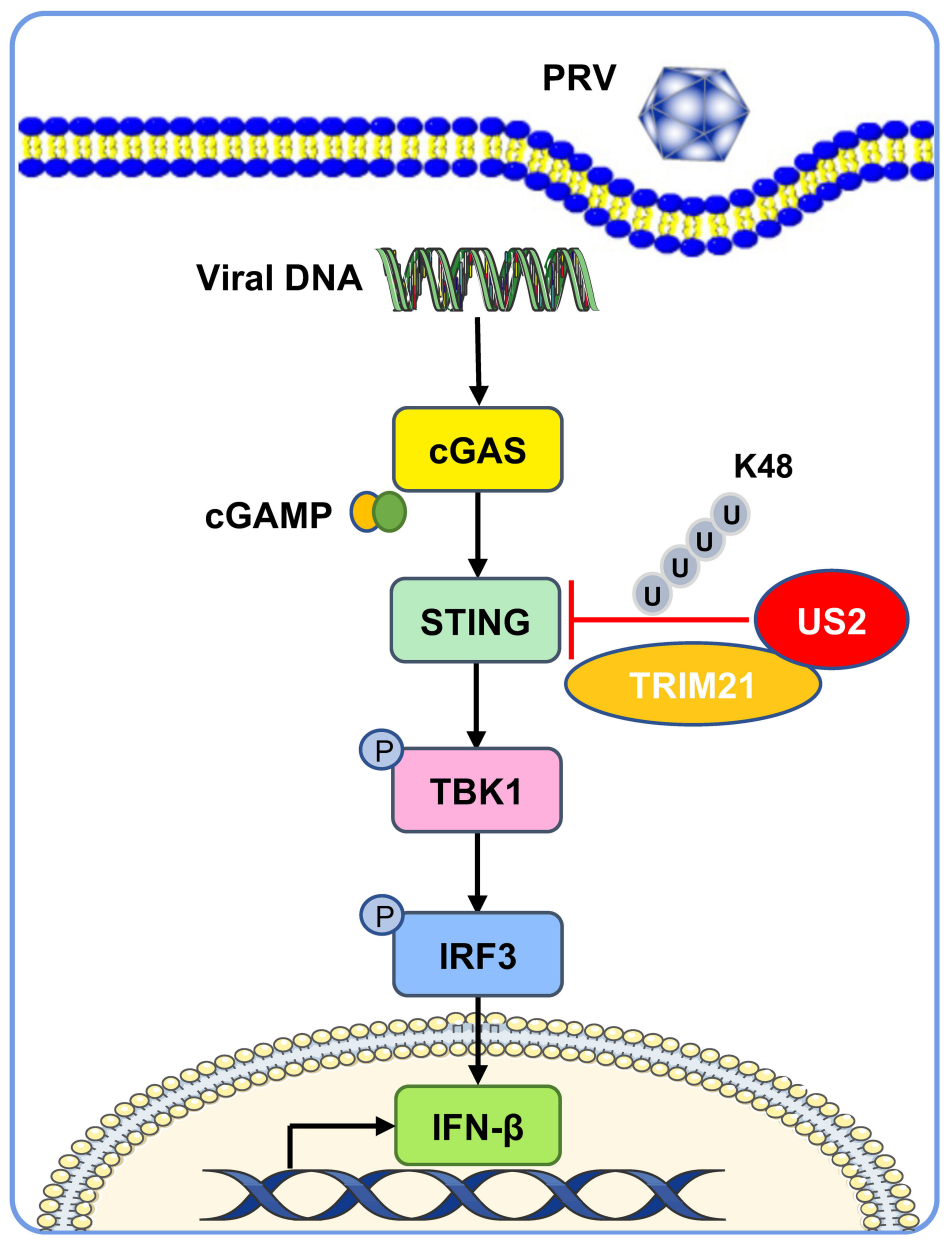
A working model of how US2 regulates the STING-mediated immune response. During PRV infection, the tegument protein US2 binds to STING in the cytoplasm and recruits the E3 ligase TRIM21, which induces STING K48-linked ubiquitination and degrades STING via the proteasome pathway. This strategy inhibits STING signaling and impairs host antiviral immune responses.

The cGAS-STING-induced IFN response promotes an antiviral state that restricts viral replication, a key target of viral immune evasion (30). Herpesviruses can establish long-term latent infections critical for immune evasion (31). Various herpesvirus family members disrupt the type I IFN response, an intrinsic part of their infection process (32). Our study demonstrates that PRV US2 antagonizes STING-mediated expression of IFN and downstream antiviral proteins, highlighting the importance of US2 in immune evasion as a potential antiviral target (**Figure 9**).

Pseudorabies is an epidemic disease that damages the swine industry, and the Bartha-K61 vaccine strain began to be used in China in 1970, effectively controlling the epidemic of porcine pseudorabies. However, at the end of 2011, PRV variant strains that cause swine pseudorabies spread over many Chinese regions, severely harming the pig farming industry’s bottom line (33). Compared with the classical vaccine strain, PRV variant strains are more pathogenic. Bartha-K61 vaccine provides complete protection against infection with the classical strain but only partially against infection with PRV variant strains (34). Compared to other PRV strains, the Bartha-K61 strain lacks four genes: gE, gI, US2, and US9 (35). Since herpesviruses are a class of immunosuppressive viruses, we hypothesized whether these four genes play a role in evading the immune response by the PRV variant. gE and gI are critical virulence genes for PRV (8, 36). The PRV glycoprotein gE/gI complex has been demonstrated to suppress the activation of the ERK signaling pathway, which prevents the generation of type I IFN (37). It has also been reported that PRV gE promotes CBP ubiquitin degradation and inhibits cGAS-STING-induced IFN production (38), suggesting that gE is involved in immune escape from PRV, which is consistent with our hypothesis. It has been reported that PRV-encoded tegument proteins are participating in immune escape (39). Subsequently, we focused on US2 and US9 proteins. Surprisingly, luciferase results showed that US2 significantly inhibited the activity of IFN and ISRE promoters. US2 expression reduces STING protein expression and impairs host defenses against viral invasion by DNA viruses or cellular DNA. On the other hand, a lack of US2 promotes STING-mediated signaling and boosts the transcription of IFN and antiviral genes downstream of STING. Our discovery thus illustrates a novel method by which US2 reduces innate antiviral responses by focusing on the stability of STING proteins, pointing to US2’s critical involvement in evading the cGAS-STING-mediated signaling pathway.

The α herpesvirus family remarkably conserves the tegument protein US2 (40). According to reports, PRV US2 binds to ERK and prevents ERK from being translocated to the nucleus (41), demonstrating that US2 might control the host immunological reaction. Treatment of cells with U0126, a specific inhibitor of ERK activation, resulted in a significant delay in viral release from infected cells. Also, the results showed that both ERK and Us2 activities are required for efficient PRV replication (42). It was shown that PRV US2 deletion enhances viral titers in PCCS (porcine cerebral cortex primary culture system) (43). In our study, we found that the replication capacity of both viruses is similar in PK-15 cells (porcine kidney-derived cell line), but mutant viruses have a lower replication capacity at early stages than wild-type viruses on 3D4/21 cells. This may be due to the induction of a stronger immune response by the US2 deletion virus. Recent research has demonstrated that viruses with US2-deficient and gE/gI-deficient genes cause pDC to secrete more IFNα (44), suggesting that US2 might regulate antiviral immune responses. Consistently, in our results, US2-deficient PRV induced stronger immune responses in 3D4/21 cells, and the virus was less pathogenic in mice than wild-type PRV. The homologous US2 encoded by HSV-2 functions as a ubiquitin-binding protein and binds ubiquitin molecules (45), but whether PRV US2 has a similar biological function is unclear. In our findings, US2 can induce ubiquitination of STING, but we did not test whether US2 directly ubiquitinates STING. Meanwhile, mass spectrometry and immunoprecipitation results showed that US2 recruits the E3 ligase TRIM21. US2 can be involved in protein ubiquitination, but we have not found whether US2 functions as an E3 ligase.

STING is an integral receptor in the cytoplasm, and its biological function is regulated by various post-translational modifications, including phosphorylation and ubiquitination (46). Multiple E3 ligases are involved in the regulation of ubiquitination of STING. For example, the E3 ubiquitin ligases TRIM32 and TRIM56 have been reported to promote ubiquitination of the STING-K63 linkage and facilitate STING activation (47, 48). The E3 ubiquitin ligases RNF5, RNF90, and TRIM29, on the other hand, are in charge of controlling the stability of the STING protein, and they primarily encourage polyubiquitination of the STING-K48 linkage for STING protein destruction (49–51). Here, we show that E3 ligase TRIM21 is involved in the protein degradation of STING. US2 interacts with TRIM21, and the knockdown of TRIM21 resulted in a significant attenuation of US2-induced ubiquitination of STING, demonstrating that the PRV-encoded tegument protein US2 promotes STING protein degradation by recruiting TRIM21 for immune escape. The lysine at position 150 of STING can form four different ubiquitination modifications and is the most distinctive site of STING (48, 49, 52, 53). In our results, three lysine sites (K236, K347, and K370) were identified for US2-induced ubiquitination degradation of STING. Our finding showed that the lysine at position 150 of STING did not form ubiquitination, which is different from previous reports and indicates the complexity of the biological function of STING.

TRIM21 is an E3 ligase with diverse functions that regulate the natural immune response. For example, TRIM21 induces DDX41 ubiquitin degradation and reduces DDX41-STING-mediated immune responses (54). Similarly, TRIM21 inhibits STING-IRF3-mediated immune responses during HSV-1 infection and significantly reduces IRF3 phosphorylation (55). It has been reported that STING recruits TRIM21 to induce IFI16 degradation, avoiding excessive immune responses (56). In addition to its ubiquitin degradation function, TRIM21 mediates K63 ubiquitination of IRF3 and reduces IRF3 protein stabilization (57). TRIM21 negatively regulates the DNA signaling pathway and also down-regulates the RLR signaling pathway. DHCR24 recruits TRIM21 to mediate the K27 ubiquitination modification of MAVS and inhibits its activation (58). These studies suggest that TRIM21 is a negative regulator of the immune response. In this study, we focused on the regulation of STING by US2. Whether US2 recruitment of TRIM21 regulates DDX41 and IFI16 can be further investigated.

Vaccines are currently a cost-effective method of controlling PRV infection in China (59). It is crucial to investigate new PRV vaccines because the traditional vaccination strain Bartha-K61 does not offer full protection against newly emerging PRV variants. Our results showed that US2-deficient viruses infected mice with lower viral loads in the organs and were less pathogenic to mice, suggesting that US2 is a target for novel PRV vaccines. Multiple *in vitro* passages of the PRV variant strain have been demonstrated to reduce it, and the virus has altered to lose gE, gI, US2, and US9. The mutated PRV variant strain protects against classical and emerging virulent PRVs in young piglets (60). These suggest that *in vitro* passaging attenuation is a strategy for constructing novel PRV vaccines and that US2 is a candidate gene for PRV vaccines.

In conclusion, our results suggest that US2 down-regulates the antiviral immune response by targeting STING and promoting PRV immune escape. The identification of US2 contributes to understanding the PRV immune evasion mechanism and identifies potential targets for developing novel PRV vaccines or antiviral drugs.

## Data availability statement

The data presented in the study are deposited in the ProteomeXchange Consortium via the PRIDE partner repository with the dataset identifier PXD053353.

## Ethics statement

Ethical approval was not required for the studies on humans in accordance with the local legislation and institutional requirements because only commercially available established cell lines were used. The animal study was approved by Henan Agricultural University Animal Care and Use Committee. The study was conducted in accordance with the local legislation and institutional requirements.

## Author contributions

ZK: Conceptualization, Data curation, Funding acquisition, Investigation, Methodology, Writing – original draft. XC: Data curation, Investigation, Methodology, Writing – original draft. LG: Methodology, Writing – original draft. LW: Methodology, Writing – original draft. YFZ: Data curation, Methodology, Writing – original draft. KG: Methodology, Writing – original draft. WY: Methodology, Writing – original draft. YK: Methodology, Writing – original draft. XL: Methodology, Writing – original draft. YHZ: Data curation, Writing – original draft. YD: Data curation, Writing – original draft. AS: Data curation, Writing – original draft. GQZ: Data curation, Writing – original draft. JZ: Writing – review & editing. BW: Funding acquisition, Writing – original draft. GPZ: Funding acquisition, Supervision, Writing – review & editing.
