# Supplementary material for: Pseudorabies virus tegument protein US2 antagonizes antiviral innate immunity by targeting cGAS-STING signaling pathway
Source: Front Immunol. 2024 Jul 2;15:1403070. doi: 10.3389/fimmu.2024.1403070 (PMC11250390; doi:10.3389/fimmu.2024.1403070)
Supplement: Supplementary file 1 [file Table_1.docx]

**S1 Table: Primers used in this study.**

| **Gene** | **Forward sequence (5’–3’)** | **Reverse sequence (5’–3’)** |
| --- | --- | --- |
|  | **Primer sequences for regular qPCR** | |
| p*GAPDH* | TACACTGAGGACCAGGTTGTG | TTGACGAAGTGGTCGTTGAG |
| p*IFNb1* | TGCATCCTCCAAATCGCTCT | ATTGAGGAGTCCCAGGCAAC |
| p*MX1* | GCTTTCAGATGCTTCGCAGG | TGTCGTATGGCTGATTGCCT |
| p*ISG56* | TCCGACACGCAGTCAAGTTT | TGTAGCAAAGCCCTGTCTGG |
| p*STING* | CTGCTGCTGTCCTGCTACTT | TTGCAGAGACTTCAGCTGGG |
| PRV *US2* | GGAGTGGTCCTCCGTCATC | GGACCCGCGCGAACAT |
| PRV *gD* | CACGGAGGACGAGCTGGGGCT | GTCCACGCCCCGCCTGAAGCT |
|  | **Primer sequences for regular PCR** | |
| PRV *US2* | ATGGGGGTGACGGCC | CTAGGAGATGGTACA |
